# Differentiating spillover: an examination of cross-habitat movement in ecology

**DOI:** 10.1098/rspb.2023.2707

**Published:** 2024-02-14

**Authors:** Rachel R. Harman, Tania N. Kim

**Affiliations:** Department of Entomology, Kansas State University, 123 W. Waters Hall, Manhattan, KS 66506, USA

**Keywords:** community structure, dispersion, edge effect, colonization, emigration, movement behaviour

## Abstract

Organisms that immigrate into a recipient habitat generate a movement pattern that affects local population dynamics and the environment. Spillover is the pattern of unidirectional movement from a donor habitat to a different, adjacent recipient habitat. However, ecological definitions are often generalized to include any cross-habitat movement, which limits within- and cross-discipline collaboration. To assess spillover nomenclature, we reviewed 337 studies within the agriculture, disease, fisheries and habitat fragmentation disciplines. Each study's definition of spillover and the methods used were analysed. We identified four descriptors (movement, habitat type and arrangement, and effect) used that differentiate spillover from other cross-habitat movement patterns (dispersal, foray loops and edge movement). Studies often define spillover as movement (45%) but rarely measure it as such (4%), particularly in disease and habitat fragmentation disciplines. Consequently, 98% of studies could not distinguish linear from returning movement out of a donor habitat, which can overestimate movement distance. Overall, few studies (12%) included methods that matched their own definition, revealing a distinct mismatch. Because theory shows that long-term impacts of the different movement patterns can vary, differentiating spillover from other movement patterns is necessary for effective long-term and inter-disciplinary management of organisms that use heterogeneous landscapes.

## 1. Introduction

The movement of individuals is a critical ecological process that influences local and regional population sizes, stability and species coexistence [1,2]. The concept of cross-habitat movement of propagules dates to the 1960s with the work by McArthur and Wilson concerning island biogeography to describe the movement of organisms across habitats to promote biodiversity, gene flow and population persistence [3]. The magnitude of movement can affect populations and communities through altered gene flow, colonization and rescue events and thus impact mating, interspecific competition, niche partitioning and resource use. However, characterizing and quantifying movement patterns in ecology remains a challenge.

Cross-habitat movement is a process that can be broken down into three different stages from the perspective of the moving individual. The first stage includes the purpose for emigrating from the donor patch into the recipient patch. Several mechanisms may influence emigration, such as the pressure to search for resources, mates or refuges; the need to escape from competition, disturbance and predation; as well as changes in landscape structure (e.g. habitat quality of either habitat, edge permeability, size and shape, [4,5]). Thus, non-random inter-habitat movements are started by either a push from a donor habitat or a pull into a recipient habitat [6].

The second stage is the movement pattern itself, including what direction the individual goes, the distance travelled, and if the individual returns or not. For example, both the theoretical and empirical literature model differences in movement, such as comparing movement to a correlated random walk [7] or measuring tortuosity [8]. Similar abiotic and biotic mechanisms impact the choices (e.g. direction, speed, whether to feed or remain stationary) that individuals make while moving [5]. Movement may change temporally with seasons or changes within vegetation/community structure (e.g. the movement of pollinators when a valuable resource is available, [9]). Long-term temporal changes on movement decisions are expected to occur with many systems under climate change [10].

The third stage is any effect of the moving individual on the recipient environment or community. Effects due to movement in the recipient habitat may include habitat modification through foraged vegetation or trail creation (e.g. via landscape modifiers such as elephants, [11]). The movement of one organism may impact the gene flow of others, such as changes in genetic neighbourhood due to pollinator movement [12], and long-term community changes through predation, competition, and population change can arise with changes in population density or novel colonization [13].

**Figure 1.**
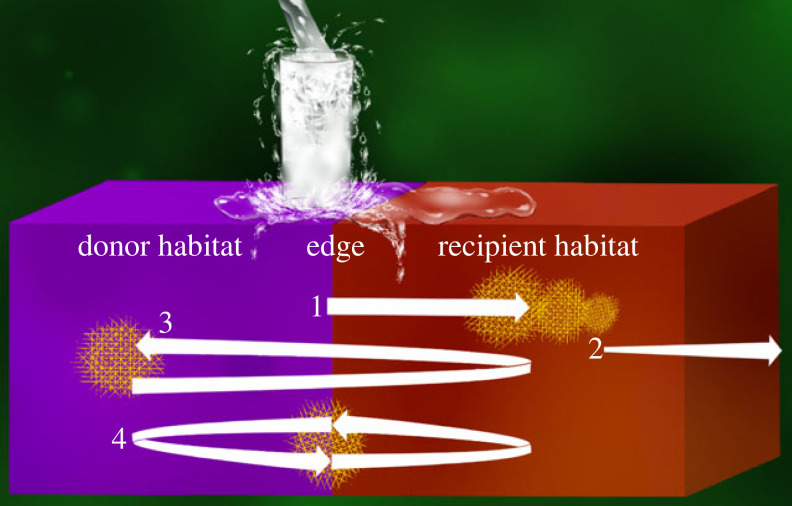
Visual of the four cross-habitat movement patterns that generally occur within the second stage of the cross-habitat movement process, including: 1) spillover, 2) dispersal, 3) foray loop and 4) continuous edge movement. Length and width of arrows indicate the expected relative distance of a moving organism and the effect size, respectively. Yellow hatching represent locations where populations are likely to establish. Spillover is additionally portrayed as a cup spilling over with water.

While many studies have examined stage 1 (factors affecting leaving, [14,15]) and stage 3 (factors affecting colonizing, [16,17]), fewer studies have examined stage 2 (movement patterns, [5]). Because movement patterns can determine the type of and magnitude of effect that is inflicted upon the recipient habitat (stage 3, e.g. the movement of grasshoppers on the vegetative structure impacted by herbivory, [18]), understanding and quantifying movement patterns in spatially explicit ways are essential for understanding the consequences of the movement process for ecological communities [19]. Here, we discuss four main movement patterns including dispersal, foray loops, continuous edge movement and spillover (figure 1).

### (a) Four cross-habitat movement patterns

Dispersal is a successive, multistep movement process of emigrating from one habitat into a different habitat, through which the organism transfers, and then finally settles into a third habitat [20,21]. Dispersal can occur as an active or passive attempt at leaving a natal or breeding site to relocate into a different breeding site [20]. Often, the intervening transferred, recipient habitat is of poor quality and termed the matrix, which can negatively impact dispersing individuals due to changes in quality and length, and thus connectivity between habitats [4,22]. Dispersing organisms having little or no influence on the recipient matrix habitat, which they primarily just transfer through [23] and are not likely to reproduce within, potentially leading to a decrease in lifetime fecundity (e.g. Glanville fritillary butterfly moving within hostile fields, [24]). Dispersal has broad-scale implications and is often modelled at the landscape scale [25] as metapopulations and metacommunities are maintained through the dispersal process [26]. The spatial scales are organism dependent, and habitats may include geographically distant habitats, such as distant forests (e.g. Euglossine bees moving through pastures 5 to 30 km in length between Brazilian Atlantic Forest fragments, [27]) or close individual trees (e.g. epiphytic lichen inhabiting single trees in Sweden, [28]). However, for each case, there is movement within a different, less hospitable environment that is between the two hospitable habitats.

Another cross-habitat movement pattern that differs from dispersal are foray loops or searches. This type of movement strategy occurs when the organism searches for a resource (e.g. food [29], mate [30] or preferred habitat [31] outside of its resident habitat or territory), but eventually returns to the resident habitat (common for central place foragers such as bees and birds). This movement pattern has been particularly recognized in fragmented and agricultural landscapes where habitat edges and mixed habitat types are prevalent. Foray searches in increasingly larger ellipsoidal loops can be an effective method for organisms to detect a suitable habitat to immigrate into through dispersal; however, there can be costs of movement, including mortality, associated with searching [32]. Theoretically, foray search strategies are often short-distanced with limited time in the adjacent, recipient habitat and, consequently, produce spatially smaller metapopulations than a directionally motivated strategy like a biased-correlated random walk [31,33]. Although movement for foraging or pollination often influences the adjacent recipient habitat, the range of the effect is greatest closest to the edge [34]. For example, pollinating bee communities have been noted to travel limited distances into agricultural fields before returning to their home habitat [35].

A third movement type involves mobile species whose home ranges overlap both habitats where individuals will continuously move between them and along habitat edges. Species with continuous movement patterns may take advantage of temporally limited resources in either habitat, creating predicable spatio-temporal dynamics [36]. Species may exhibit continuous movement patterns, which may be difficult to distinguish from others without understanding their life history. Many species are attracted to edge environments that have complementary resources provided by different habitats or the occurrence of rare resources from either habitat [37,38]. For instance, lemurs may reside near or move to forest edges to opportunistically use plant resources [39]. Variability in resources is a primary reason why richness is often higher along the edges of fragmented habitats (e.g. carabid beetles, [40,41]). The distribution of the edge-dwelling populations are impossible to differentiate from other movement patterns as densities and ecological impact are often greater near the edge than further into either habitat [42,43]. However, continuously moving edge populations are not likely to spread further into the habitats as individuals return to the edge or to the adjacent habitat.

Finally, the term ‘spillover’ has been used to generally describe cross-habitat movement but has varied meanings depending on study field, and thereby lacks a clear definition. ‘Spillover’ was first introduced in economics as an impact that seemingly unrelated events in one nation can have on the economies of other nations [44]. In the 1960s, physicists used spillover to describe the movement of particles across boundaries (e.g. hydrogen spillover, [45]). Both fields describe spillover as one-way or unidirectional movement readily depicted as a cup spilling over with water (figure 1). However, the term was not used by ecologists until the 1980s as an explanation for enhanced biodiversity in waters adjacent to and beyond marine protected areas [46]. Researchers in the disease discipline picked up the term in the 2000s to describe transmission of infectious agents from reservoir animal populations [47]. Since the word entered the biology field, several authors have translated spillover differently, often with meanings that overlap other movement patterns. This likely has correspondingly led to variation in the methods used to measure spillover and in the interpretation of spillover consequences for populations and communities. We argue that a clearer definition of ‘spillover’ is needed to distinguish it from the other movement patterns. A definition that aligns with the original use of spillover [44,45,48] would avoid such confusion.

The consequences of the four movement patterns vary for both the moving individuals and the communities in the recipient habitat, yet many studies do not distinguish between these movement patterns and often use them as synonyms. For example, foray loops and random dispersal are often used interchangeably yet the directionality and spatial extent of these movement patterns are different [32]. Specifically, dispersing individuals have little impact on the transient recipient habitat but would have greater impacts on the final habitat where establishment occurs. In contrast, individuals moving with a looping pattern may impact the recipient habitat, but their effects on their own populations remain in the donor habitat. Similarly, the effects of individuals moving continuously at the edge are often confounded with the effects of spillover (*sensu* [48]). Because continuously moving individuals remain along habitat edges, their effects may be felt along the edges of both habitats. By contrast, because individuals moving via spillover will eventually establish into the recipient habitat, their effects on the recipient habitat are consistent and have long-term implications compared with the other three movement patterns described above. While all four movement patterns describe cross-habitat movement, the lack of clear definitions and interchangeable use of terms is problematic. This is especially important in applied disciplines where long-term management solutions and practices are needed to either promote or prevent cross-habitat movement from occurring in the case of conservation or invasion spread, respectively.

**Figure 2.**
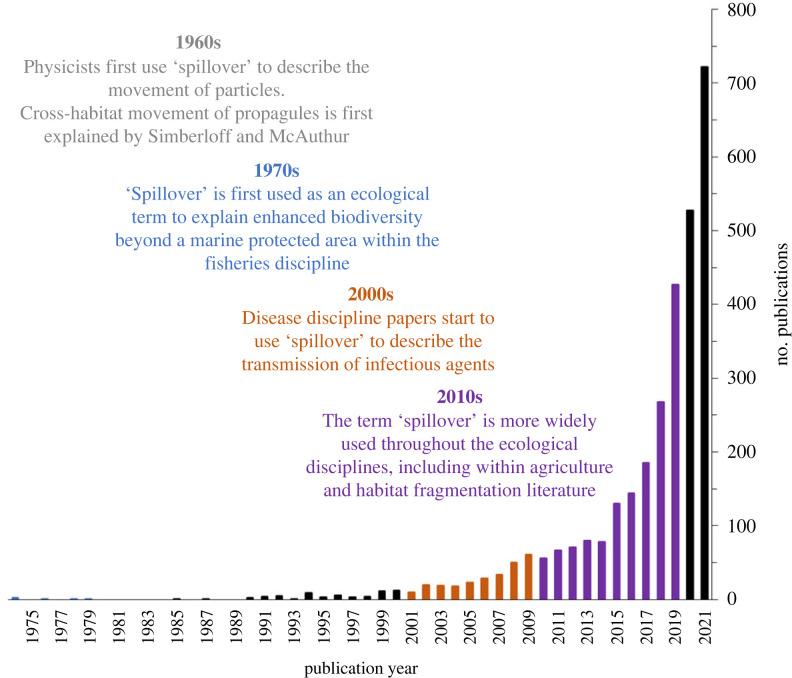
Histogram of the number of ecology-based Web of Science categories published each year with the keyword search of ‘spillover’.

In this systematic review, we describe how these four movement patterns are used within the literature and we further distinguish ‘spillover’ as a distinct movement pattern with important population and community consequences. The term spillover is widely used in several applied ecological disciplines and has increased dramatically over the last few years (figure 2). Although a few reviews have already been published on the spillover processes, these primarily look at 1) the consequences of movement rather than focus on the movement pattern itself and 2) are focused on a single system (e.g. marine [49] and insects in agricultural systems [50,51]). Thus, we examine empirical ecology-based papers including those related to the fisheries, disease, agriculture and habitat fragmentation disciplines. Our objectives are to: 1) analyse definitions and methods used to define spillover through a systematic literature review, 2) describe a definition of spillover that is unique to the other three movement patterns based on definitions in Objective 1 and our expert input, and 3) detail the potential implications of the specific definition, including suggestions for methods that would allow the different movement patterns to be measured.

## 2. Objective 1: a systematic review of ‘spillover’

### (a) Methods of the systematic review

We compiled a database of spillover studies found in the Web of Science (http:www.webofknowledge.com) records up to 31 March, 2021. We used the search terms 'spillover’ and ‘spill over’. We then excluded categories outside the biology discipline (e.g. economic or chemical spillover) and human epidemiology literature, which uses language very distinct from ecological work. We further refined our search using ‘organism’, ‘biodiversity’ or ‘population’. Articles included in our database: 1) specifically mentioned ‘spillover’ or ‘spill over’ within the text, 2) used living organisms as the propagule of movement (thus excluding nutrients and sediment) and 3) empirically assessed spillover or the consequences of spillover. We did not include articles that used a similar concept to spillover to describe the allochthonous flow or spatial subsidies of nutrients and sediment across adjacent habitats [52] because we focused on propagules that can replicate to have long-lasting effects on recipient habitat and because they did not explicitly use the term spillover. Including other terminology related to spillover (but did not directly use the term spillover) would have created biases and redirected the study's focus since this paper focused on understanding how scientists used the spillover term. This way, we collected a broad spectrum of papers that included ecological, behavioural, evolutionary, applied and basic science articles.

Our search yielded 196 empirical papers, several of which were included more than once within the review as data for more than one system (e.g. different communities, species, methods used or habitats) were included. We treated each of these case studies as an independent replicate in our analysis for a total of 337 empirical studies (see electronic supplementary material, appendix A). For each study, the definition of spillover was analysed and grouped based on major descriptors, such as movement direction or landscape type. We noted what methods were used to measure spillover and if these methods matched with the article's own definition. Methods used to match the definition included an assessment of an organism's movement direction, path or origin, information on the landscape used, and any quantification of changes in an organism's behaviour (e.g. predatory, herbivory, mating, pollinating, settling). The studies were also categorized based on study type (observational/experimental), taxonomic group, spatial or organizational scales (habitat/landscape and individual/population/community movement, respectively), edge type (hard/soft edge dependent on the change in resources, such as expected nutrient level or vegetation type) and response measurement (e.g. movement, abundance, diversity). Lastly, to evaluate whether the proportions of the definition and methods differed, we used separate Pearson's χ^2^ tests for independence with Monte Carlo simulations of 10 000 iterations. Differences between the number of descriptors were analysed using ANOVA in R version 4.0.5 [53].

### (b) Outcomes of the systematic review

#### (i) Disciplines

The studies neatly fell into four different disciplines depending on the study system: fisheries, disease, agriculture and habitat fragmentation (box 1). The statistics mentioned above were performed among study systems as well as for all of the studies as a whole. Separating the studies in this way allowed us to quantify if these disciplines studied spillover differently. The percentage of the 337 studies placed into each discipline was 33% for agriculture, 19% in disease, 18% in fisheries and 30% in habitat fragmentation. Fifty-eight of the studies were placed into two categories.

Box 1. **Description of each discipline (bold text) that historically use 'spillover'. Example definitions from each of the disciplines are provided. Definition descriptors include: effect, habitat type, habitat arrangement and directionality, which are labelled with supercaptions 1–4, respectively.**

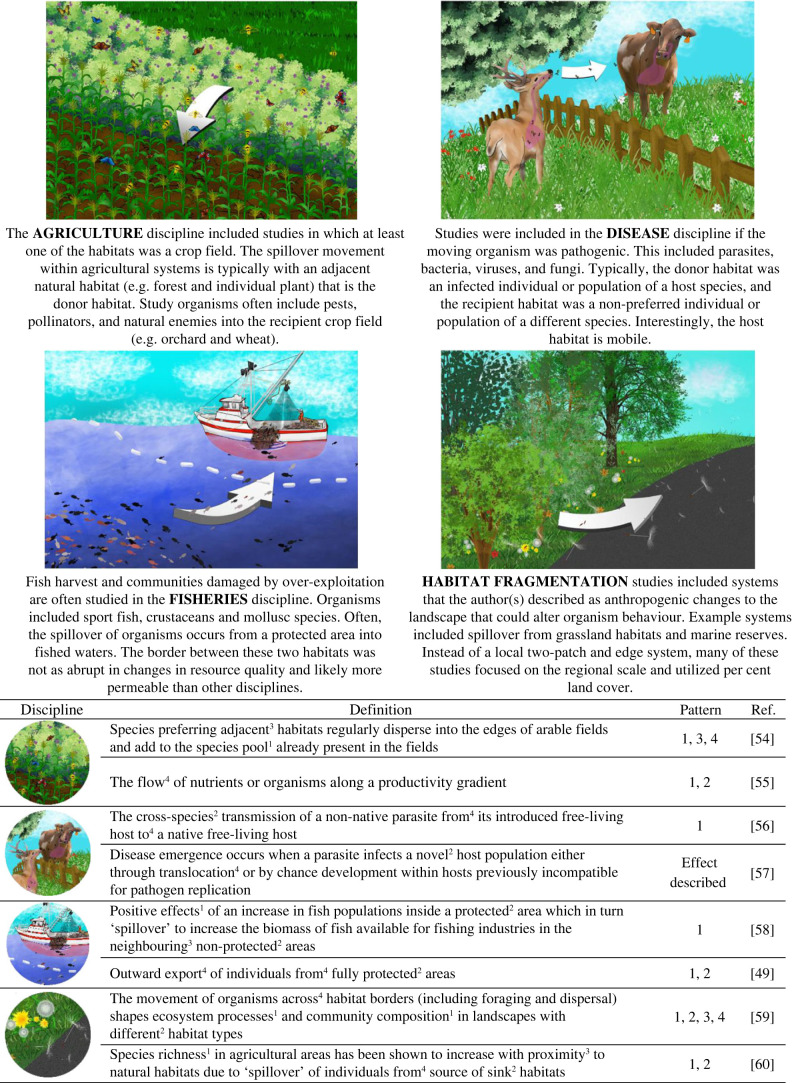

#### (ii) Breakdown of the definitions

**Figure 3.**
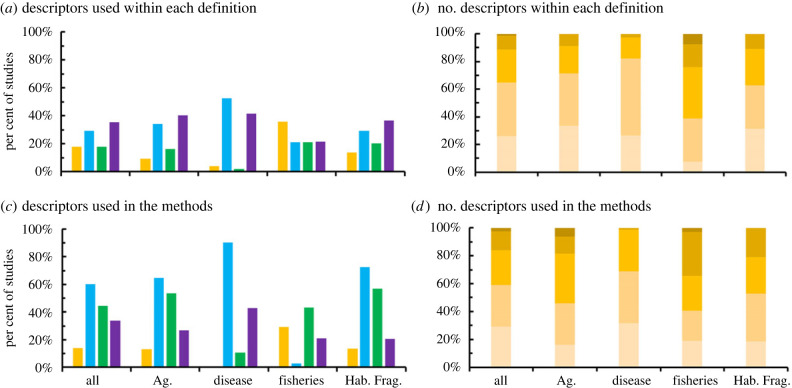
Studies that used a descriptor within their spillover definition (top) and methods for assessing spillover (bottom). The definition descriptors included directionality (gold) into a different (blue), adjacent (green) habitat with a measurable effect (purple). The proportion of total studies that included 0 to 4 descriptors (designated by lighter to darker gold in (*b*) and (*d*).

Overall, the ‘spillover’ definitions incorporated four general descriptors detailing: 1) an effect within the recipient habitat (e.g. enhanced pollination services within the recipient), 2) differences or similarities in habitat types, 3) arrangement of the landscape (e.g. donor and recipient habitats are adjacent) and 4) the directionality of movement (e.g. unidirectional from donor to recipient). Only 54% of the studies included a clear definition of spillover. The remaining 46% included the term spillover, often as a reason or consequence for the study's results. Overall, most studies used only one (43%) or two (26%) descriptors to define spillover, and a large portion (33%) of studies did not include any. Only 2% of papers included all four descriptors (table 1). This may be due to different descriptors used among disciplines (χ_212_ = 105.36, *p* < 0.001, figure 3). Studies within the fishery discipline had a clearer definition of spillover with, on average, 1.5× more descriptors than the other disciplines (*F*_4_ = 16.46, *p* < 0.001).

**Table 1.** Breakdown of descriptors used within spillover definitions (unidirectional, different habitats, adjacent habitats and effect) and possible corresponding movement patterns (spillover, dispersal, foray loop and continuous edge movement).

definition descriptor(s)	movement pattern(s)	per cent of studies (%)
unidirectional	spillover, dispersal	6
different habitat	spillover, foray, continuous	17
adjacent habitat	spillover, foray, continuous	3
effect	spillover, dispersal, foray, continuous	18
unidirectional, different	spillover	2
unidirectional, adjacent	spillover	2
unidirectional, effect	spillover, dispersal	3
different, adjacent	spillover, foray, continuous	2
different, effect	spillover, foray, continuous	8
adjacent, effect	spillover, foray, continuous	9
unidirectional, different, adjacent	spillover	3
unidirectional, adjacent, effect	spillover	0
unidirectional, different, effect	spillover	1
different, adjacent, effect	spillover, foray, continuous	3
unidirectional, different, adjacent, effect	spillover	2
none	spillover, dispersal, foray, continuous	22

#### (iii) Descriptor 1: effect

The methods used within the studies were predominately observational and nearly all studies measured a consequence of moving individuals for the recipient habitat (96%; table 1), such as a greater number of individuals [61] or species [62,63] with proximity to the edge. The most commonly measured effects of spillover included changes in density (30%), species richness (25%), presence/absence of the species/individual (20%), species interactions such as predation, herbivory and pollination (8%), and diversity (5%) between recipient and donor habitats. For disease studies, following the movement path of viruses and many other pathogens would be logistically challenging or impossible, and thus presence or differences of immune response were used. Despite the high prevalence of a spillover effect, most studies could not differentiate whether these results were indeed due to spillover or whether they were generated from other movement patterns.

#### (iv) Descriptor 2: habitat type

Of the reviewed studies, 38% of definitions clearly stated that the habitats in the landscape were different, as in an abiotic (e.g. nutrient) or biotic (e.g. predation pressure or vegetation type) change in habitat that impacted the species. Empirical studies often incorporated habitats with distinctly different resource types (59%) such as food (e.g. spillover of bees into agricultural fields from a non-crop habitat, [64]) or shelter resources (e.g. spillover of rabies from skunks to domestic animals, [65]). An additional 21% of studies differentiated habitats by trophic interactions, such as predation rate (e.g. outside of a marine protected area used within the fisheries discipline, [66]).

To properly assess movement between different habitats, it is necessary to collect data from both habitats to determine if organisms return to the donor habitat or move randomly throughout the landscape. However, only 67% of studies tested the habitats on both sides of the edge. For instance, Barros *et al*. [67] used pairwise point counts of sights and vocalizations of forest birds to determine bird diversity in forests and pasture habitats. Similar sight counts are often used in fishery studies, of which 81% collected data in both habitats. In addition to collecting data from both the donor and recipient habitats, methods should also include assessments within both habitat cores, and not limited to movement along edges. These data will provide information as to whether cross-habitat movement is a result of the edge or a standard behaviour.

#### (v) Descriptor 3: habitat arrangement

Of all the studies, 22% included adjacency as part of their definition of spillover. Most empirical papers did have two adjacent habitats as the arrangement, although only 43% sampled along the habitat edge, and often assumed cross-habitat movement. Half of all studies used a hard boundary between the habitats, which we labelled as an abrupt change in resources, such as between forest and agricultural fields [68] or two different host species [69]. As such, 98% of fisheries papers included a soft boundary, which was created by the change in management (i.e. marine protected areas to unprotected fished waters), a human-constructed zone that a fish may not perceive. Although both soft and hard boundaries may include spillover processes, the movement behaviour at the border between habitats will differ in these two habitat types as organisms are more likely to cross a soft, graduated border than an abrupt one [38]. For the disease discipline, we accounted for other terms to supplement adjacency, such as interactions or proximity between hosts, however, this field rarely described the spatial arrangement of the hosts (2%).

The spatial extent of the study is an important factor to consider in cross-habitat movement and a large portion of all studies (56%) assessed spillover at the local two-habitat scale while 43% of all studies examined spillover at the regional, multi-habitat scale. In particular, disease studies focused on movement at the regional spatial scale (77% of the disease studies) while the other three disciplines examined movement at the local-habitat scale (59% agriculture, 58% habitat fragmentation and 77% of fishery studies). Only 3 studies assessed movement at both scales to investigate whether the ability to identify spillover processes varied with spatial extent [70]. For instance, Kovács-Hostyanszki *et al*. [70] assessed bee abundance and pollination success between different habitats at both the local (adjacent fields) and regional (multi-habitat) scales. They found contrasting evidence of whether a spillover event was statistically perceived depending on spatial scale, and thus whether pollination services were provided to the recipient habitat.

Furthermore, studies differed in spillover effects at varying levels of organization. In particular, disease research assessed the movement of populations (77%, e.g. spillover of conjunctivitis from house finches to other bird species, [71]) but both agriculture (70%) and habitat fragmentation studies (85%) included movement of communities (e.g. spillover of 122 bird species from fragmented cloud forests to pastures, [72]). Researchers in the fishery discipline similarly assessed spillover across many levels of organization: individuals (31%), populations (41%) and communities (28%).

#### (vi) Descriptor 4: directionality of movement

The authors with clear definitions were nearly twice as likely to define spillover as a movement process (45%) rather than an effect (24%) within the habitat. Terms describing directional movement, such as ‘from’, ‘to’, ‘diffused’ and ‘directional’, were descriptors in 13% of studies. When assessing the methods used to quantify movement, 97% of the studies' sampled organisms could have arrived in the recipient habitat via any of the four movement patterns, making it unclear which movement pattern had actually occurred. Studies that used methods to distinguish unidirectional movement (the remaining 3%) often included directional traps or marking individuals using either radiotelemetry or a tag identifier. Fisheries and agriculture disciplines were highly represented in the movement-based definitions of spillover (29% and 21%, respectively). Fishery studies, in particular, define the movement as unidirectional (58% of fishery studies), compared with 9% of agricultural studies. Even within these two disciplines, however, methods that assess for directionality are less common (16% and 24% in agricultural and fisheries disciplines, respectively).

Importantly, the criteria that we used to discern if directionality was tested were strict. Only methods that would directly show direction or movement path were noted as showing direction, as this would measure if individuals were returning to the donor habitat or not. The majority of studies used methods that measured a change possibly due to movement processes (e.g. changes in abundance, richness, herbivory; see Descriptor 1 section). Fifty-six per cent of studies in agriculture, fisheries and habitat fragmentation included data measured at varying distances from the edge into the recipient habitat. For example, Moseby *et al*. [73] examined the effects of fences surrounding a conservation reserve on the activity of wildlife species, and measured counts of footprints and trapped animals at varying distances from the fenced edge. The authors concluded that positive spillover effects occurred because densities gradually decreased outside of the conservation area (in the recipient habitat), however, they did not directly measure directionality, and thus these studies were also not included within the ‘directional movement’ group.

The lack of movement data may be rooted in the over-simplification of movement behaviour and the complex methods needed to fully measure it [19]. Movement is often accepted as linear, a hypothesis that promotes simpler measurements of displacement (e.g. flight mills) to be extrapolated as continuous movement [54,74]. Additionally, it is difficult to track organisms continuously; these methods can be limited in both study system and effectiveness. For example, large-bodied organisms (e.g. fish and deer) are often tracked because of easy telemetry attachment; however, due to the expense of using these tools, there are fewer replicates than with simple directional traps [75,76]. For smaller organisms or passive dispersers such as insects and plants, movement pattern can be assessed visually and is often done in simplified landscapes to allow for easier tracking [77,78]. Continual improvements in tracking technology will allow for more precise descriptions of movement patterns for a more comprehensive array of organisms.

#### (vii) Overall mismatch of definition and methods used

We evaluated whether the methods used to assess spillover, matched each study's own definition of spillover. We considered a mismatch of definition and methods if, for example, a study's definition of spillover included the movement of an organism across an edge (Descriptor 4) to an adjacent, different habitat (Descriptors 3 and 2) but their methods did not actually quantify movement. Measuring an effect (Descriptor 1) was considered if a direct impact on the habitat was quantified, such as herbivory, a negative reaction in a host, trail creation. Methods that measured habitat type included data collection within both habitats so that a comparison could be made if the habitats were similar or not. The descriptor of habitat arrangement was quantified by data collection at the edge between the habitats. Lastly, measurements of directionality consisted of the use of traps, observations, or data collection procedures that either produced a movement path or separated individuals moving in different directions. We acknowledge that these methods used in the analysis do not encompass every aspect of the descriptors, thus we could have missed studies that did measure a descriptor differently.

**Figure 4.**
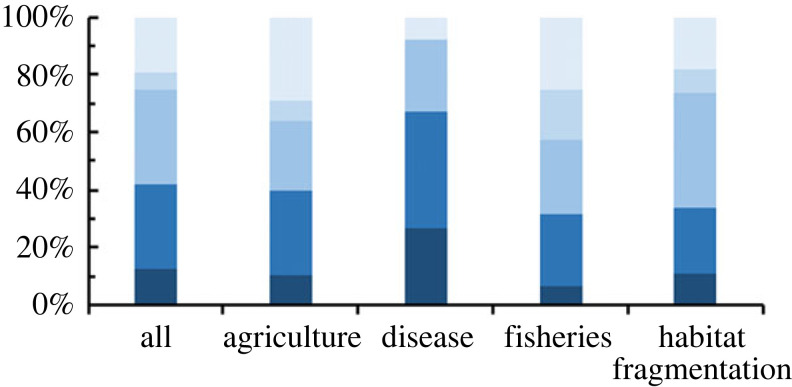
Per cent of all studies within research discipline that used methods corresponding to their own definition based on four descriptors (movement direction, habitat type and arrangement, and effect). Darker colour indicates a substantial match between the study's definition and the methods used to quantify it.

Given this, there was great mismatch between spillover definitions and methods used. Specifically, only 12% of studies included methods that matched their own definition of spillover ([79,80]; figure 4). Of the remaining studies, 84% of the papers included directionality within the definition but did not include methods to measure directionality. Likewise, a great proportion of papers did not include measurements within different habitat types (58%), adjacent habitats (57%) or a direct effect to the recipient habitat (69%) even though these descriptors were stated within their definitions of spillover. Finally, 20% of studies used methods that quantified completely different descriptors than those stated in their definition.

The matching of methods to the definition descriptor was significantly different among the disciplines (*χ*_216_ = 96.63, *p* < 0.001). Disease and habitat fragmentation studies generally incorporated methods that quantified the descriptors from their definitions (50% and 52% of disease and habitat fragmentation studies, respectively; figure 4), but this is likely due to the fewer number of descriptors used in the definition itself. Even so, habitat fragmentation and agricultural studies often stated that there would be an effect in the recipient habitat but did not measure one directly (92% and 85% of habitat fragmentation and agricultural studies, respectively). Studies within the fisheries discipline had the greatest mis-match of all the disciplines (68% of fisheries studies); however, their definitions included significantly more descriptors. Interestingly, studies that both defined and measured spillover with unidirectionality was within the fisheries discipline, possibly due to the broader use of telemetry data [81] and a more standardized spillover definition within the discipline.

## 3. Objective 2: a definition of spillover

We extracted four general descriptors from each study's own definition of spillover. In order to distinguish spillover from other cross-habitat movement patterns (foray loops, dispersal and continuous movement), specific descriptors are needed. The first descriptor (an effect caused by a moving organism) is expected to occur with all four movement patterns, thus this descriptor alone is not sufficient to designate a spillover event. The second descriptor (habitat type) considers if the habitats are similar or different, as measured by changes in abiotic (e.g. nutrient) or biotic (e.g. predation pressure) conditions, and include spillover, foray loops and continuous edge movement as these movement types occur at the edge between two different environments. In contrast, the emigration and transfer stages of dispersal occur at the edge of different habitats; however, the effect of the moving organism likely impacts the final habitat where the organisms establish and is more similar in quality to the donor habitat than the adjacent habitat. The third descriptor (habitat adjacency) is necessary for spillover movement. Dispersal includes a three-habitat system, two of which are separate, and for continuous or foray movements, adjacency is not necessary and can occur at larger landscape scales. Lastly, the direction of movement separates directional movement patterns, such as spillover and dispersal, from movement patterns where the moving organisms return to the donor patch (i.e. foray loops and continuous movement).

Using a combination of these four major descriptors, each movement pattern can be succinctly and distinctly defined (figure 1). Specifically, spillover can be defined as:

the unidirectional movement from a donor habitat to a different, adjacent recipient habitat that is affected by the organism.

Our definition is not novel and follows the same principles of spillover used by Gardes *et al.* [45] and Russ and Alcalca [48]. It showcases spillover as a specific process and is created by unifying definitions provided by authors of previous spillover review papers. For instance, Rand *et al*. [50] defined spillover as ‘the movement of subsidized natural enemies across agricultural-to-natural habitat edges'. Blitzer *et al*. [51] used the definition of ‘movement that results in the function of an organism no longer being fulfilled in the habitat where the organism comes from but in the habitat where the organism moves to’. Within disease ecology, Faillace *et al*. [82] defined spillover as the ‘introduction of a pathogen from a primary host into a susceptible secondary host’, with host habitats being two different species. Lastly, Di Lorenzo *et al*. [49] defined spillover as the ‘export of individuals… through the active movement of juveniles, subadults and adults' outwards from a marine protected area. While these previous definitions are similar to ours, they do not separate from other movement patterns, unlike the one we propose (table 2 for definition of all movement types using the descriptors). Additionally, because the definition proposed here is not limited to any specific discipline or system, it will allow for better within- and cross-discipline collaboration. We leave it to the experts to determine what is appropriate for each descriptor for their system.

**Table 2.** Definitions for each of the four movement types defined using the four definition descriptors obtained from reviewed literature. Definition descriptors included effect, habitat type, habitat arrangement and directionality, which are labelled with supercaptions 1–4, respectively.

movement pattern	definition using descriptors
dispersal	The unidirectional^4^ movement of an organism from one habitat to another^2^ using a three-stage process that includes emigration, transfer through a matrix^3^ and immigration. This process generates gene flow^1^
foray loop	Systematic^4^ exploration/foraging^1^ strategy between^3^ habitats that concludes when the organism returns^4^ to the original^2^ habitat
constant	Continuous^1^ movement within the organisms' home range^3^, consisting of one or more different habitats^2^. This process does not create gene flow^4^ as populations generally do not overlap
spillover	The unidirectional^4^ movement from one donor habitat to a different^2^, adjacent^3^ recipient habitat that is affected^1^ by the moved organism

## 4. Objective 3: potential implications of the specific definition

### (a) Need for specific nomenclature

The lack of differentially defining spillover from other movement patterns likely contributes to the lack of consistency among and within biological disciplines. These ambiguous definitions have led to several problems including a mismatch of study definitions to the methods used to measure them. We call for a more precise and unifying definition of spillover as understanding the impacts of anthropogenic disturbances become more interdisciplinary and communication and collaboration are required for environmental and social solutions. As much of this understanding is driven by theory, a narrow definition would also be useful to model outcomes of spillover events and to collect empirical data for model parameterization.

Differentiating the four movement patterns is important as each pattern will result in different dispersion patterns (i.e. placement of individuals/populations in space) as establishment or recolonization will occur in different locations within the two-habitat system (figure 1). Spillover is the only pattern with colonization fully within the adjacent recipient habitat and these organisms are likely to spread further into the recipient habitat through density-dependent factors. Organisms spilling over thus have great influence on the adjacent, recipient habitat that, if populations persist and move, can be long-reaching and continuous through time. Consequently, differentiating spillover from other movement patterns is necessary for effective long-term management of organisms that utilize heterogeneous landscapes, which is particularly relevant in human-modified landscapes due to climate change, reserve creation [73], habitat change or loss [83], as well as human-introduced invasive species [84]. For example, in agriculture, predictions of natural enemy spillover distances from natural refuges can be overestimated if the movement is looped instead of linear [85]. This would promote the use of intermittent hedge rows instead of border crops to promote beneficial insect abundances [35,86]. For diseases, the difference between a spillover event from one host to an individual of a non-preferred host species or a dispersal event in which the recipient host can readily infect individuals of the same species determines the difference between a carrier [71] and a potential outbreak [87].

An additional challenge from spillover studies is that the long-term implications for the moving propagules are not clear. Different population-level dynamics and spatial assortments can arise with different individual movement patterns ([88], box 1). For example, in metapopulations, unidirectional source-sink movement can lead to varying levels of population persistence compared with frequent, continuous movement of a panmictic population [2]; similar outcomes can be observed for describing cross-habitat movement. Once spillover is clearly defined, spatially explicit models can be created and used to understand the long-term and large-scale consequences of spillover for populations and communities, especially for species conservation, pest and invasion control, and slowing disease spread [89,90]. Finally, because limiting the effects of human-mediated disturbance requires cross-discipline action, utilizing similar terminology would promote collaboration and avoid confusion.

### (b) Suggestions for future research

The lack of a clear definition has led to a distinct mismatch of the methods used to quantify an ambiguous term. The majority of empirical work that tests for spillover includes measurements of a spillover effect or outcome (e.g. changes in abundance, richness, habitat damage between recipient and donor habitats) rather than spillover as a process. Studies within the fisheries discipline tended to have more specific definitions and included movement measurements, such as those gained through telemetry data (e.g. distance moved, orientation, [76,91]) This may be due to this field using the term for longer than the other fields, allowing for more universal nomenclature (figure 2).

As spillover is often defined as a movement process, methods that actually measure the movement of organisms should be included in future studies to differentiate spillover from other movement patterns. Such methods may consist of mark-recapture, telemetry and GPS tracking, all of which measure both the distance and trajectory of movement over time (for more examples, table 3). Tests should be performed in different, adjacent habitats with data collected at the border as well as within the cores of both habitat types to serve as a control. Additional methods can be used to obtain information to gauge which movement pattern is occurring, even if the path itself is unknown. For instance, using pit-fall traps or sticky cards that collect organisms moving in only one direction can separate spillover from foray movement [86,116]. Lastly, assessing variables that likely trigger the spillover movement (i.e. density, resource limitation, mate scarcity, competition) would provide much needed information that is lacking in the current empirical literature. Understanding the causes of movement would allow us to better predict the consequences of spillover movement in landscapes and promote better management and conservational strategies.

**Table 3.** Comparison of methods, potential limitations and example studies that do or do not measure spillover based on each definition descriptor.

descriptors		methods	potential limitations	reference examples
unidirectional	yes	Radiotracking individuals	Expensive with few replicates; often large distance between towers; limited information at the population or community level; limited to large-bodied organisms	Encarnacao *et al.* [92]; Pittman *et al.* [93]
		Traps that collect organisms moving from one direction	Measures activity density, not relative density; limited to organisms that can be trapped	Birkhofer *et al.* [94]; Schneider *et al.* [59]
		Mark-release-recapture	Unidirectionality can be assessed, but other non-linear movements are difficult to differentiate if there are few recapture events	Follesa *et al.* [95]; Gray *et al.* [96]
		Release and visual tracking of individuals or populations	Unidirectionality can be assessed, but other non-linear movements are difficult to differentiate if done en masse	Abesamis and Russ [97]; Sperry *et al.* [98]
	no	Data collection along transects	Assumes that location represents linear movement away from the border	Boinot *et al.* [63]; Chakraborty *et al.* [99]
		Pairwise counts of sights or vocalizations	Often does not incorporate distance from edge; assumes location represents linear movement away from the border	Barros *et al.* [67]; Thomine *et al.* [85]
		Genetic analysis for relatedness	Assesses overall gene flow, not the potential for return; generally used for dispersal studies, not spillover	Bianco *et al.* [100]; Teske *et al.* [101]
different	yes	Collecting data in two different habitat types	Need to have two habitat types in which movement can occur between	Estavillo *et al.* [68]; Ng *et al.* [102]
	no	Collecting data from one habitat	Assumes that the presence of organisms originated from the other habitat; no control to make comparisons with	Alos *et al.* [103]; Casini *et al.* [104]
		Collecting data from multiples of the same habitat type	Often does not incorporate distance from edge; assumes that location represents linear movement away from the border	Almberg *et al.* [75]; Rizali *et al.* [105]
adjacent	yes	Using adjacent habitats	Need to have two habitat types next to each other in which movement can occur between	Abecasis *et al.* [76]; Catton *et al.* [106]
		Collecting data from the border between the habitats	There needs to be a clear border or zone between the habitats; data collection may be limited due to either area or arrangement type	Boetzl *et al.* [107]; Picchi *et al.* 108]
		Using multiple scales to assess spillover processes	Additional data collection can provide more information than assessing one scale alone	Kovacs-Hostyanszki *et al.* [109]
	no	Using spatially separated habitats	Includes a matrix or other type of habitat that changes the movement behaviour of an organism	Almberg *et al.* [75]; Bowman *et al.* [110]
		Incorporating landscape type (e.g. % land cover) within the model	Assumes the presence/abundance of organisms is due to the spillover from another habitat; increases the chance of having soft boundaries that create different movement patterns than hard edges	Delogu *et al.* [111]; Munoz-Saez *et al.* [112]
effect	yes	Quantifying a change in the recipient habitat	Changes may not be dependent on the spillover movement; changes may be difficult to measure	Almberg *et al.* [75]; Clem and Held [113]
		Quantifying a change in behaviour of the organism	Behaviour is difficult to quantify; behaviour may be dependent on other variables	Birkhofer *et al.* [94]; Dobbs and Potter [114]
		Quantifying a change in population or community structure	Often done instead of quantifying movement; changes may not be dependent on the spillover movement	Abesamis and Russ [97]; Peralta *et al.* [80]
	no	Not addressing a change	Cannot differentiate from transitory movement behaviour and more permanent or influential movement	Afonso *et al.* [115]

Although these methods mentioned above are ideal for obtaining movement path data, we concede that different systems may have to individualize methods for their specific needs. For instance, small arthropods cannot be as easily tracked as larger mammals due to the size of tracking devices and the movement restrictions these devices can cause. Additionally, other marking techniques, such as etching and the use of colored paint or powder, is not feasible with microbes. Therefore, molecular techniques that might indirectly infer movement, such as environmental DNA, gut content analyses, genetic testing or stable isotopes [117–120], hold promise for smaller organisms or those with cryptic behaviours. For example, performing genetic tests to see the relatedness of populations can separate spillover from dispersal movement, but not foray or edge movement, if regional metapopulations are included in the assessment [100]. Furthermore, microcosm-based research can be used to predict the consequences of movement for smaller organisms in artificial landscapes. Microcosms have been commonly used to examine the movement of small invertebrates [121,122] and provide information about the system that would be difficult to obtain in the field.

Finally, because spillover is a process, the temporal scale of this movement pattern needs to be considered. Field measurements conducted at within-generation time-scales could be misleading and be perceived as one type of movement pattern instead of another at longer-temporal scales, such as with cyclic colonization [123]. For example, soybean aphids require two hosts throughout their seasonal cycle. In the winter, aphids mate and overwinter in buckthorn along forest edges but then move into adjacent soybean in the spring to feed and asexually reproduce [124]. Assessments of soybean aphid movement within a season would likely indicate a spillover pattern; however, if examined across seasons, the species is continuously returning to its donor habitat and utilizes both resources. Understanding if the soybean aphid species will continuously spread through spillover or return to the donor habitat is key to management success. Therefore, researchers should consider time scales that are appropriate for their study system and research question.

## 5. Conclusion

Spillover is described as a distinct ecological process with important consequences separate from other well-established movement terms, such as forays and dispersal. Despite this, little has been published about these potential differences as few papers dismantle the movement patterns from one another. Incorporating similar methods to quantify spillover and other movement patterns among the applied disciplines will additionally open doors for better communication and collaboration. Interdisciplinary work is essential in ecology, and clear language will promote spillover research of global issues such as viral pandemics, climate change, food security and habitat depletion.

## Data Availability

Data are available for private download through the Dryad reviewer https://doi.org/10.5061/dryad.sbcc2fr8v [125]. Supplementary material is available online [126].
